# The prevalence of urinary tract infections in type 2 diabetic patients: a systematic review and meta-analysis

**DOI:** 10.1186/s40001-022-00644-9

**Published:** 2022-02-05

**Authors:** Nader Salari, Mohammad Mahdi Karami, Shadi Bokaee, Maryam Chaleshgar, Shamarina Shohaimi, Hakimeh Akbari, Masoud Mohammadi

**Affiliations:** 1 Department of Biostatistics, School of Health, Kermanshah University of Medical Sciences, Kermanshah, Iran; 2 Student Research Committee, Kermanshah University of Medical Sciences, Kermanshah, Iran; 3 Faculty of Health and Life Sciences, School of Life Sciences, Coventry University, Coventry, UK; 4 Department of Biology, Faculty of Science, University Putra Malaysia, Serdang, Selangor Malaysia; 5 Cellular and Molecular Research Center, Gerash University of Medical Sciences, Gerash, Iran

**Keywords:** Type 2 diabetes, Urinary tract infection, Diabetes, Prevalence, Meta-analysis

## Abstract

**Background:**

Urinary tract infection is the most common infection in type 2 diabetic patients. Various studies have reported different outbreaks of urinary tract infections in type 2 diabetic patients. Therefore, the present study aimed to determine the prevalence of urinary tract infections in type 2 diabetic patients during a systematic review and meta-analysis in order to develop interventions to reduce the incidence of urinary tract infections in type 2 diabetic patients.

**Methods:**

In this study, systematic review and meta-analysis of study data related to the prevalence of urinary tract infection in type 2 diabetic patients were conducted using keywords including type 2 diabetes, urinary tract infection, diabetes, prevalence, meta-analysis and their English equivalents in SID, MagIran, IranMedex, IranDoc, Google Scholar, Cochrane, Embase, Science Direct, Scopus, PubMed and Web of Science (WoS) databases from 1993 to 2020. In order to perform the analysis of qualified studies, the model of random-effects was used, and the inconsistency of studies with the *I*^2^ index was investigated. Data analysis was performed with Comprehensive Meta-Analysis (Version 2).

**Results:**

Based on a total of 15 studies with a sample size of 827,948 in meta-analysis, the overall prevalence of urinary tract infection in patients with type 2 diabetes was 11.5% (95% confidence interval: 7.8–16.7%). The prevalence of urinary tract infections in diabetic Iranian patients increased with increasing number of years of research, (*p* < 0.05), and with increasing age of participants (*p* < 0.05), but however the prevalence decreased with increasing sample size (*p* < 0.05).

**Conclusion:**

This study shows that urinary tract infections are highly prevalent in patients with type 2 diabetes. Therefore, due to the growing prevalence of diabetes and its complications such as urinary tract infections, the need for appropriate screening programs and health care policies is becoming more apparent.

## Background

Diabetes is the most common endocrine disorder in the last century. In developing countries, various factors, including lifestyle changes, have increased the incidence of the disease [1]. There are two main types of diabetes, and type 2 diabetes is more common. Type 2 diabetes is a chronic and progressive metabolic disease involving a heterogeneous group of disorders associated with varying degrees of insulin resistance, insulin secretion disorder, insulin development and persistence, and increased glucose production [2–6]. The prevalence of type 2 diabetes has increased in recent years [7, 8]. In 2015, about 415 million adults with type 2 diabetes were reported, which is projected to increase to 642 million by 2040 [7]. The prevalence increased from 4.3% to 9% in men and 5% to 7.9% in women [8]. Type 2 diabetes increases the risk of certain diseases, including cardiovascular disease, eye and blindness problems, amputation of the lower limbs, kidney disease and infectious diseases [9, 10]. The most common infectious disease in diabetic patients is type 2 urinary tract infection (UTI) [11]. It is estimated that 150 million people worldwide suffer from urinary tract infections each year [12].

A urinary tract infection (UTI) is an infection of the urinary system. This type of infection can involve the urethra (a condition called urethritis), kidneys (a condition called pyelonephritis) or bladder (a condition called cystitis) [10–12], Women are at greater risk of developing a UTI than are men, Infection limited to the bladder can be painful and annoying. However, serious consequences can occur if a UTI spreads to the kidneys [11, 12]

Factors such as immune system disorders, weakening of white blood cells, poor blood supply, bladder dysfunction due to nephropathy and glucosuria can cause urinary tract infections in type 2 diabetic patients [13–22]. Dysuria is a complication of urinary tract infection in diabetic patients due to organ damage and even death due to the complexity of pyelonephritis. Also, these patients experience urinary retention, urgency, and incontinence during the night due to increased urination to excrete excess glucose [23]. The prevalence of urinary tract infections in women is higher than in men, which may be due to the specific structure of the short urinary tract, the shortness of the urethra, and its proximity to the anus in women [24]. Urinary tract infections make it difficult to control blood sugar in diabetic patients, which increases the need for blood sugar monitoring, reduces the quality of life, and imposes significant treatment costs on the patient [25].

Statistics reported from various studies indicate heterogeneity in reported prevalence, indicating the inconsistency and uncertainty of the prevalence of UTI in type 2 diabetes patients. Therefore, since intervention studies on reducing the prevalence of UTI in type 2 diabetes patients require accurate and consistent information to prevent the complications of UTI, the research question is that what is the overall prevalence of UTI in type 2 diabetes patients? The findings from this study could provide a better understanding for the development of more detailed programs to reduce the effects of urinary tract infections and improve people's health.

## Methods

The study looked at the systematic review and meta-analysis to find related studies from the SID, MagIran, IranMedex, IranDoc, Cochrane, Embase, Science Direct, Scopus, PubMed and Web of Science (WoS) databases, and the Google Scholar search engine. The articles were obtained using keywords such as prevalence, urinary tract infection, type 2 diabetes and Latin keywords Prevalence, UTI, Type 2 diabetes mellitus and all possible combinations of these words the search strategy for each database was determined.

Keywords were extracted from the Medical Subject Headings (MeSH) database. Keywords related to the studied population (P) were: prevalence, outbreak, Type 2 diabetes mellitus, Type 2 diabetes, diabetes and outcome-related keywords (O) were: UTI, urinary tract infection, infection, morbidity, outcomes.

The search strategy in each database was determined by using the advanced search option and using all possible keyword combinations with the help of AND, and OR operators.

Search strategy in all databases: ((((((((Type 2 diabetes [Title/Abstract]) OR Type 2 diabetes mellitus [Title/Abstract]) OR diabetes mellitus [Title/Abstract]) AND urinary tract infection [Title/Abstract]) OR UTI [Title/Abstract]) OR infection AND prevalence OR Period Prevalence OR Point Prevalence))))))), The search strategy for all databases is reported in Table 1.

**Table 1:** Search strategy in all databases

Databases	Searching strategy
Google Scholar	Diabetes "Type 2 diabetes" "Type 2 diabetes mellitus" prevalence outbreak urinary tract infection "cross sectional"
SID, MagIran, IranMedex, IranDoc	prevalence of UTI in type-2 diabetes patients
Cochrane, Embase	((((((((Type 2 diabetes [Title/Abstract]) OR Type 2 diabetes mellitus [Title/Abstract]) OR diabetes mellitus [Title/Abstract]) AND urinary tract infection [Title/Abstract]) OR UTI [Title/Abstract]) OR infection AND prevalence OR Period Prevalence OR Point Prevalence)))))))
PubMed	(Type 2 diabetes [MeSH]) AND urinary tract infection) [Title/Abstract] OR diabetes [Title/Abstract] OR Type 2 diabetes [Title/Abstract] OR Type 2 diabetes mellitus [Title/Abstract] OR diabetes mellitus [Title/Abstract] AND urinary tract infection [Title/Abstract] OR UTI [Title/Abstract] OR water infection [Title/Abstract]
Science Direct	diabetes OR Type 2 diabetes OR Type 2 diabetes mellitus OR diabetes mellitus AND urinary tract infection OR UTI OR Infection
Scopus	Type 2 diabetes mellitus "AND" urinary tract infection "OR "Type 2 diabetes" OR "Type 2 diabetes mellitus "OR" diabetes mellitus" AND "urinary tract infection" OR "UTA"
WOS	TI = (Type 2 diabetes OR Type 2 diabetes mellitus OR diabetes mellitus) AND TI = (prevalence OR outbreak) AND TI = (urinary tract infection OR UTI)

All related studies were identified in the search process, and the information of these studies was transferred to the information management software (EndNote). Therefore, all possible related articles published from 1993 to 2020 were identified, and their information was transferred to EndNote. In order to maximise the comprehensiveness of the search, the list of sources used in all related articles found in the above search was manually reviewed.

### Inclusion criteria

Criteria for entering studies included: studies that examined the prevalence of urinary tract infections in type 2 diabetic patients, descriptive studies, cross-sectional studies, observational studies and studies in which the full text was available.

### Exclusion criteria

Criteria for excluding studies include intervention studies, case studies, case–control, cohort, grouping, review and irrelevant studies, studies without sufficient data, repeatability of studies, and uncertainty of study methods. Because the prevalence studied in this study is population-based, only cross-sectional studies were included in the study and other studies such as the case–control and cohort studies, which may report the prevalence but were group-based, were not included in the study.

### Selection of studies

Initially, studies that were repeated in various databases searched were removed from this study. The researchers of this study then prepared a list of the titles of all the remaining articles to get obtain articles by evaluating the articles in this list.

In the first stage, screening, the title and abstract of the remaining articles were carefully studied and deleted based on the criteria for including and excluding unrelated articles. In the second stage, i.e. the evaluation of the competence of the studies, the full text of the possible related articles remaining from the screening stage was examined based on the inclusion and exclusion criteria, and in this stage, the unrelated studies were removed.

To prevent bias, all stages of investigation and search were performed based on the PRISMA process by 3 researchers; all sources were reviewed and extracted by two researchers independently. If the articles are not included, the reason for deleting them was mentioned. In cases where there was a disagreement between the two researchers, a third person would review the article. Twenty studies entered the third stage, qualitative assessment.

### Qualitative evaluation of studies

In order to validate and evaluate the quality of articles (methodological validity and results), a checklist appropriate to the type of study is used. STROBE checklists are commonly used to critique and evaluate qualitative observational studies such as the present study. The STROBE checklist consists of 6 scales or general sections including title, abstract, introduction, methods, results, and discussion. Some of these scales have subscales for a total of 32 items. These 32 items represent different methodological aspects of the study, including title, problem statement, study objectives, study type, study statistical population, sampling method, appropriate sample size determination, the definition of variables and procedures, study data collection tools and methods which includes statistical analysis and findings. Accordingly, the maximum score obtained from the qualitative evaluation will be in the STROBE 32 checklist, and considering the score of 16 as the cut-off point, the articles with scores of 16 and above will be considered good and medium quality articles and will be included in the study. Also, 16 articles with poor methodological quality were considered weak and therefore excluded from the study [42] (Table 2).

**Table 2:** Evaluation of the quality of studies with the STROBE checklist

Row	Author (s) and year of publication	Introduction		Materials and methods								
		Title and abstract	Introduction	study method	Performing environment	Contributors	Variables	Data source and measurement method	Bias	Sample size	Quantitative variables	Statistical analysis methods
1	Muller, 2005, [9]	+	+	+	+	+	+	+ +	−	−	+ +	+
2	Fu, 2014, [11]	+	+	+	+	+	+	+ +	−	−	+ +	+
3	Carrondo, 2020, [27]	+	+	+	+	+	+	+ +	−	+	+ +	+
4	He, 2018, [28]	+	+	+	+	+ +	+ +	+ +	−	+	+ +	+
5	Nicolas, 2017, [29]	+	+	+	+	+	+	+ +	−	+	+ +	+
6	Sewify, 2016, [30]	+	+	+	+	+	+	+ +	−	+	+ +	+
7	Wilke, 2016, [31]	+	+	+	+	+	+	+ +	−	+	+ +	+
8	Al-rubeaan, 2012, [32]	+	+	+	+	+	+	+ +	−	+	+	+
9	Yu, 2014, [33]	+	+	+	+	+	+	+	−	+	+	+
10	Hiriji, 2012, [34]	+	+	+	+	+	+	+ +	−	+	+	+
11	Hammar, 2010, [35]	+	+	+	+	+	+	+ +	−	−	+ +	+
12	Janifer, 2009, [36]	+	+	+	+	+	+	+ +	−	+	+ +	+
13	Venmans, 2009, [37]	+	+	+	+	+ +	+ +	+ +	−	+	+ +	+
14	Goswarni, 2001, [38]	+	+	+	+	+	+	+ +	−	+	+ +	+
15	Bonadi, 2000, [39]	+	+	+	+	+	+	+ +	−	−	+ +	+

Row	Author (s) and year of publication	Results					Discussion and conclusion					
		Descriptive data	Participants' reports	Report the original data	The main results of the study	Other analyzes	Report key results	Limitations	Interpretation of results	Generalisation	Budget	Support
1	Muller, 2005, [9]	+	+	+	+	−	+	+	+	−	−	+
2	Fu, 2014, [11]	+	+ +	+ +	+ +	−	+	−	+ +	−	−	+
3	Carrondo, 2020, [27]	+	+	+	+	+	+	+	+ +	−	−	+
4	He, 2018, [28]	+	+ +	+	+ +	+	+	+	+ +	−	−	+
5	Nicolas, 2017, [29]	+	+ +	+	+	+	+	+	+ +	−	−	+
6	Sewify, 2016, [30]	+	+	+	+	+	+	+	+ +	−	−	+
7	Wilke, 2016, [31]	+	+	+	+	+	+	+	+ +	−	−	+
8	Al-rubeaan, 2012, [32]	+	+	+	+	+	+	+	+	−	−	+
9	Yu, 2014, [33]	+	+	+	+	+	+	+	+	−	−	+
10	Hiriji, 2012, [34]	+	+	+	+	+	+	+	+	−	−	+
11	Hammar, 2010, [35]	+	+ +	+ +	+ +	−	+	−	+ +	−	−	+
12	Janifer, 2009, [36]	+	+	+	+	+	+	+	+ +	−	−	+
13	Venmans, 2009, [37]	+	+ +	+	+ +	+	+	+	+ +	−	−	+
14	Goswarni, 2001, [38]	+	+ +	+	+	+	+	+	+ +	−	−	+
15	Bonadi, 2000, [39]	+	+	+	+	−	+	+	+	−	−	+

In the present study, based on the evaluation based on the STROBE checklist, 15 papers entered the systematic review and meta-analysis process as good and moderate methodological quality studies and five papers had poor methodological quality were therefore excluded from the study [26].

### Extracting the data

Information on all final papers entered into the systematic review and meta-analysis was extracted from a pre-prepared checklist. The checklist included the title of the article, the author's first name, year of publication, place of study, sample size, the prevalence of urinary tract infection in type 2 diabetic patients, and mean age.

### Statistical analysis

The I^2^ test was used to assess the heterogeneity of the selected studies. In order to investigate the publication error, due to the high volume of samples entered into the study, the Egger test was used at a significance level of 0.05 and the corresponding funnel plot was used. Data analysis was performed using Comprehensive Meta-Analysis software (Version 2).

## Results

In this study, systematic review and meta-analysis of data on the prevalence of urinary tract infections in type 2 diabetic patients without time constraints and according to PRISMA guidelines were systematically investigated. Based on an initial search of the database, 1904 possible related articles were identified and transferred to the information management software (EndNote). Out of a total of 1904 identified studies, 173 were duplicates and were eliminated.

**Fig. 1:**
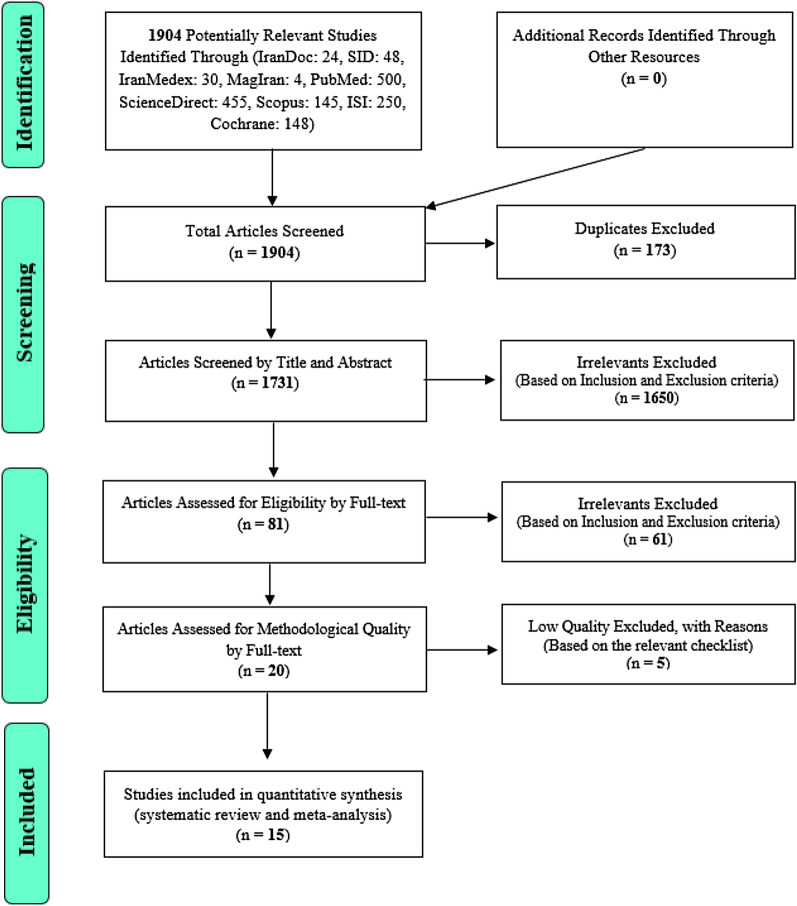
The flowchart on the stages of including the studies in the systematic review and meta-analysis (PRISMA 2009)

Out of 1731 remaining studies in the screening stage, 1650 articles were deleted by reading the title and abstract based on inclusion and exclusion criteria. In the competency assessment phase, 61 of the remaining 81 studies were eliminated by studying the full text of the article based on inclusion and exclusion criteria due to unrelatedness. In the qualitative evaluation stage, through the study of the full text of the article and based on the score obtained from the STROBE checklist, out of the remaining 20 studies, five articles that had poor methodological quality were removed. Finally, 15 studies entered the final analysis (Fig. 1).

Based on the results of the test (*I*^2^: 99.9) and due to the heterogeneity of the selected studies, the random-effects model was used to combine the studies and the common estimate of the prevalence. The total sample size was 872,948 people, and the specifications of the selected articles are given in Table 3.

**Table 3:** Characteristics of included studies on prevalence of urinary tract infection

Author, year, [References]	Mean age (year)	Country	Sample size	Study time range	Prevalence %
Muller, 2005, [9]	65.7	Netherlands	6712	1 year	6.9
Fu, 2014, [11]	56	USA	89,790	1 year (1 Jan 2010–1Dec 2010)	9.4
Carrondo, 2020, [27]	71	Portugal	7347	1 year (1 Jan–1 Dec 2015)	16.2
He, 2018, [28]	59.3	China	3264	3 years and 4 months (Mar 2013–Jul 2016)	12.5
Nicolas, 2017, [29]	60.2	USA, Germany	392,995	9 years (2006–2014)	33.3
Sewify, 2016, [30]	55.5	Kuwait	722	3 years (Apr 2011–Mar 2014)	34.9
Wilke, 2016, [31]	73.8	USA, Germany	456,586	2 years (2010–2012)	9.2
Al-rubeaan, 2012, [32]	51.9	Saudi Arabia	1000	18 years (1993–2009)	25.3
Yu, 2014, [33]	62.5	USA	73,151	3 years and 8 months (1 Jan 2008–9 Sep 2011)	8.2
Hiriji, 2012, [34]	63	USA, Germany, Sweden	135,920	1 year	2.5
Hammar, 2010, [35]	57.4	Sweden	6016	3 years (2004–2007)	2.3
Janifer, 2009, [36]	–	India	1157	1 year	42.8
Venmans, 2009, [37]	67	Netherlands	6343	1 year (2000–2002)	2.7
Goswarni, 2001, [38]	33	India	155	6 months	9.03
Bonadi, 2000, [39]	69.4	Italy	490	2 years (1996–1998)	18.1

**Fig. 2:**
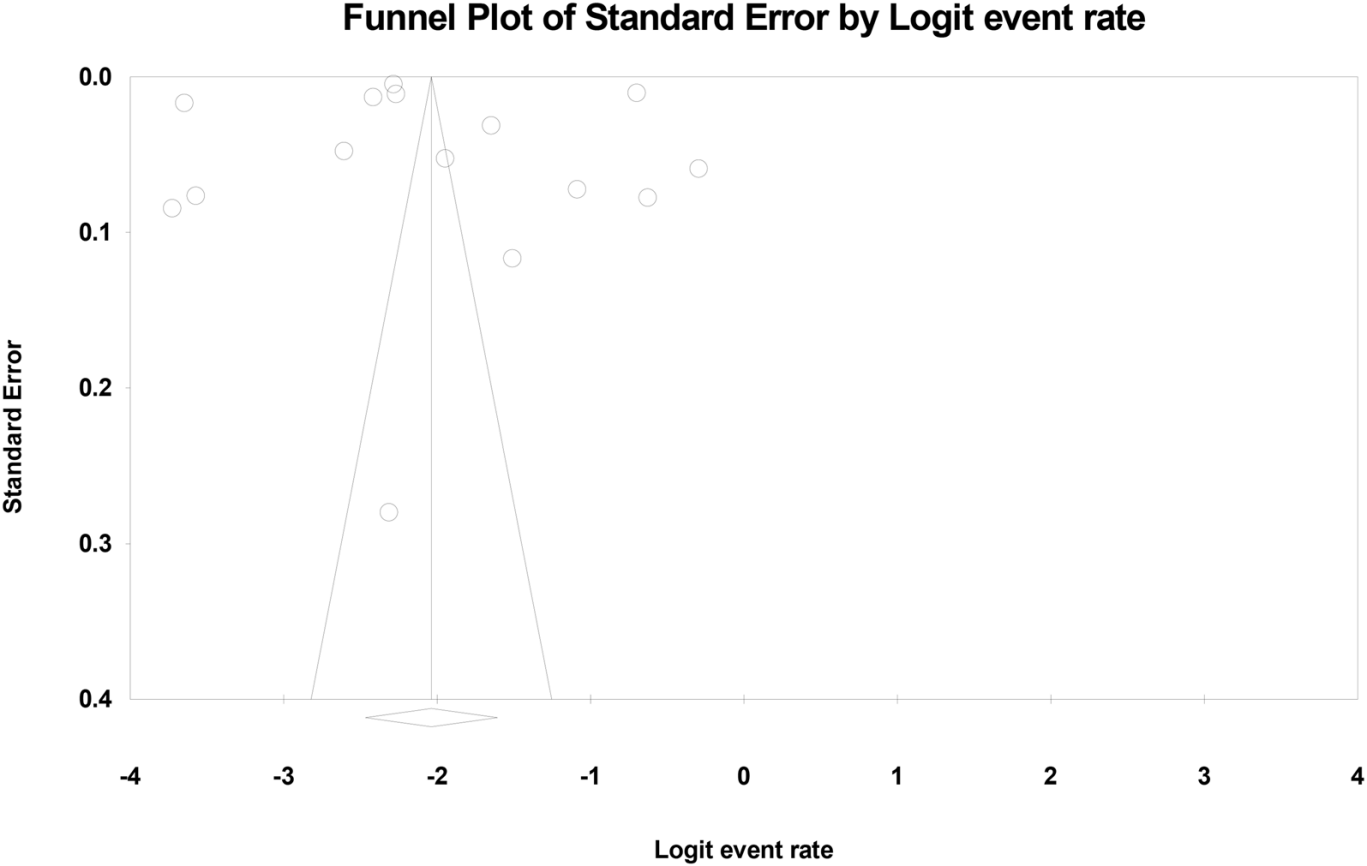
Funnel plot results from the overall prevalence of urinary tract infection in type 2 diabetic patients

The probability of publication bias in the results was investigated by funnel plot and Egger test (Fig. 2), which shows that the publication bias was not statistically significant (*p* = 0.857, also the results of Begg and Mazumdar test at the significance level of 0.1 indicate no publication bias was present in the study (*p* = 0.552).

**Fig. 3:**
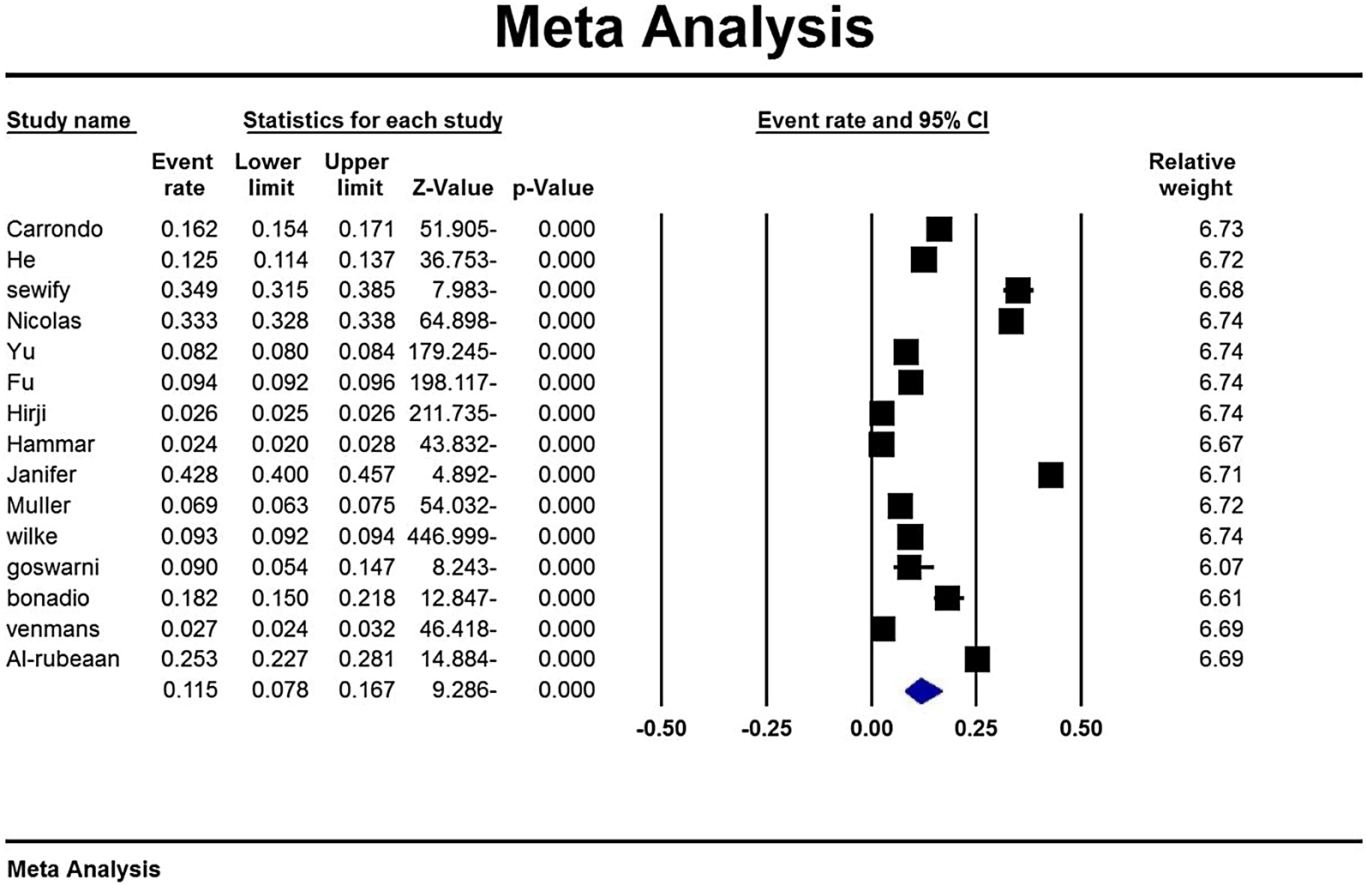
Overall prevalence of urinary tract infection in type 2 diabetic patients and 95% confidence interval

**Fig. 4:**
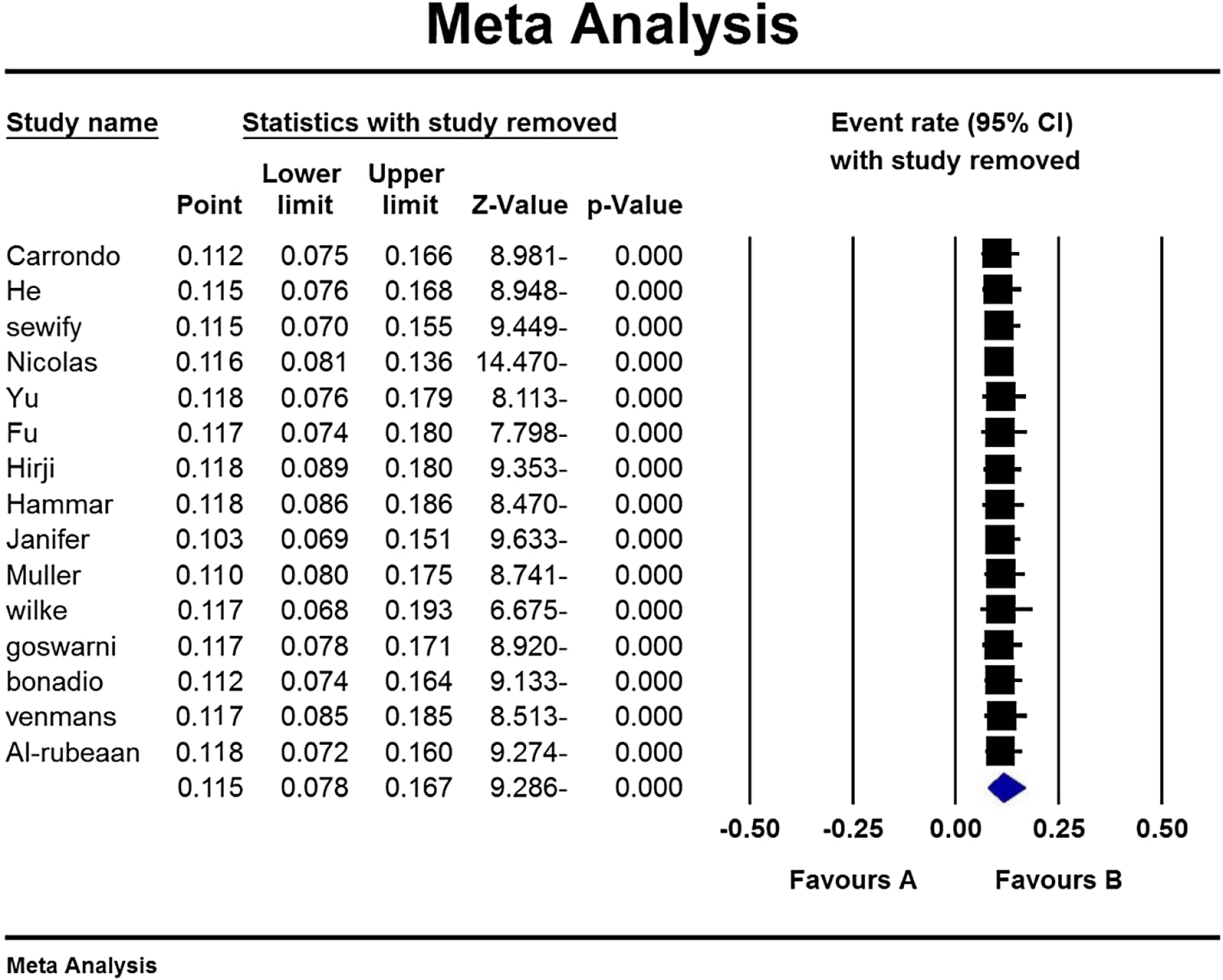
Results of sensitivity analysis

According to the study results, the overall prevalence of urinary tract infection in type 2 diabetic patients was 11.5% (95% CI: 7.8–16.7%) (Fig. 3). Due to the different time range in the studies, the analysis of the subgroup based on the studies time range is mentioned in Table 4. Also, based on the results of subgroup analysis in Table 2, it is reported that the prevalence of urinary tract infections in women with diabetes 14.2 (95% CI 9.7–20.2) is higher than men with diabetes 6.1 (95% CI 3.6–10). A sensitivity analysis was perfumed to ensure the stability results, after removing each study results did not change (Fig. 4).

**Table 4:** Analysis of the subgroup based on the studies time range

		*N*	Sample size	*I*^2^	Prevalence (95% CI)
Study time range	1 Year	7	247,424	99.9	8.9 (95% CI 4.5–16.8)
	1–4 Years	6	540,229	99.9	11 (95% CI 8.9–13.5)
	> 4 Years	2	40,290	96.4	29.3 (95% CI 22.1–37.7)
Sex	Man	14	392,835	99.9	6.1 (95% CI 3.6–10)
	Women	14	434,388	99.9	14.2 (95% CI 9.7–20.2)

The midpoint of each line indicates the prevalence of urinary tract infections in each study and the rhombic shape of the prevalence of urinary tract infections in type 2 diabetic patients for the entire study.

**Fig. 5:**
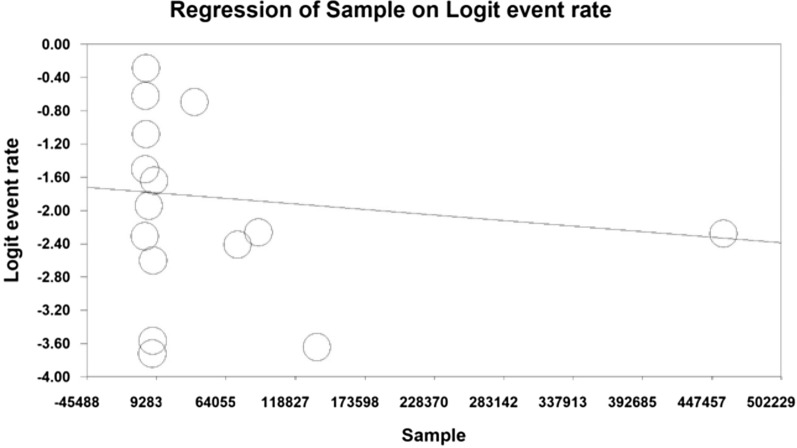
Meta-regression of the relationship between sample size and prevalence of urinary tract infection in type 2 diabetic patients

**Fig. 6:**
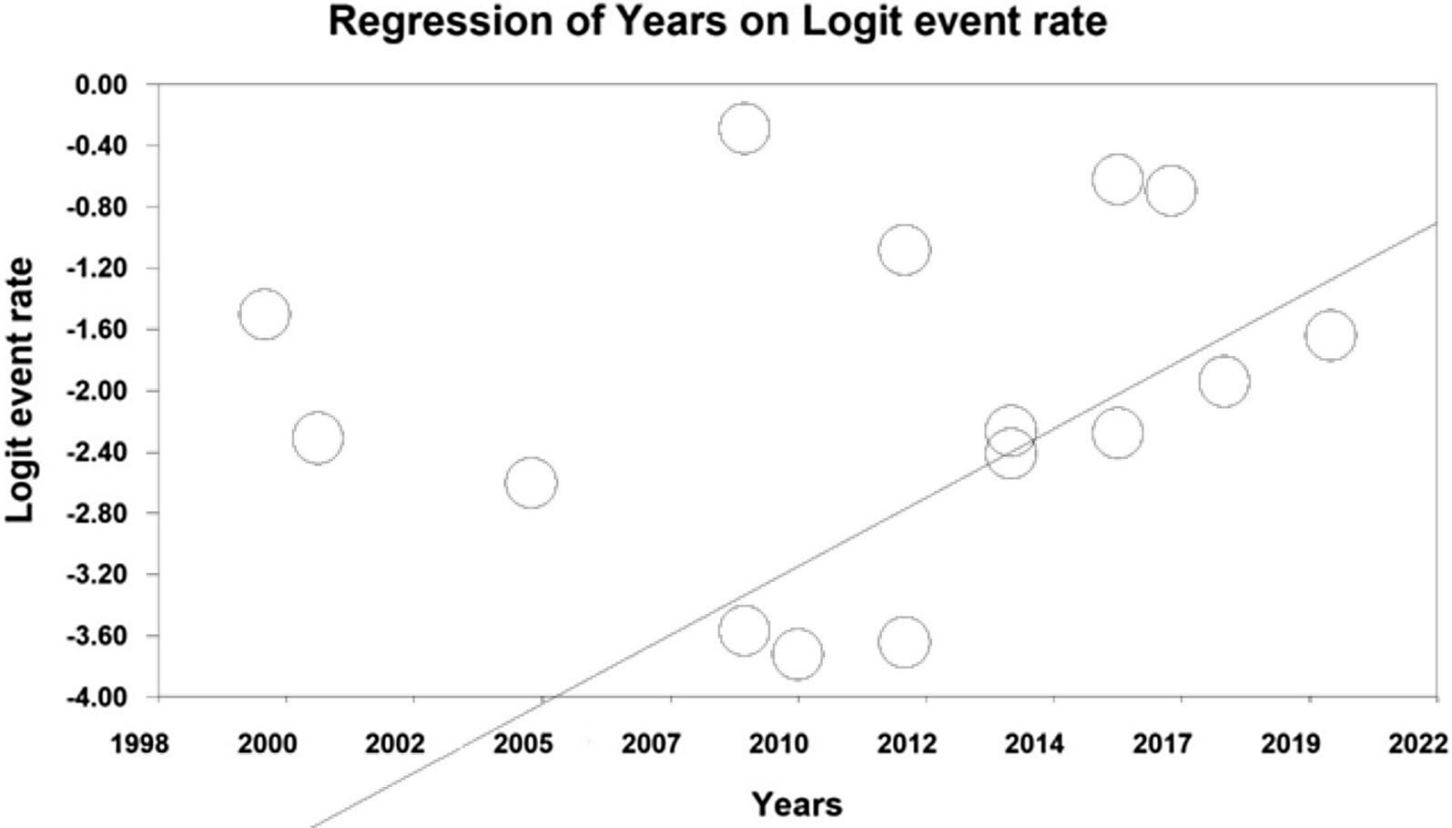
Meta-regression between the year of study and the prevalence of urinary tract infection in type 2 diabetic patients

**Fig. 7:**
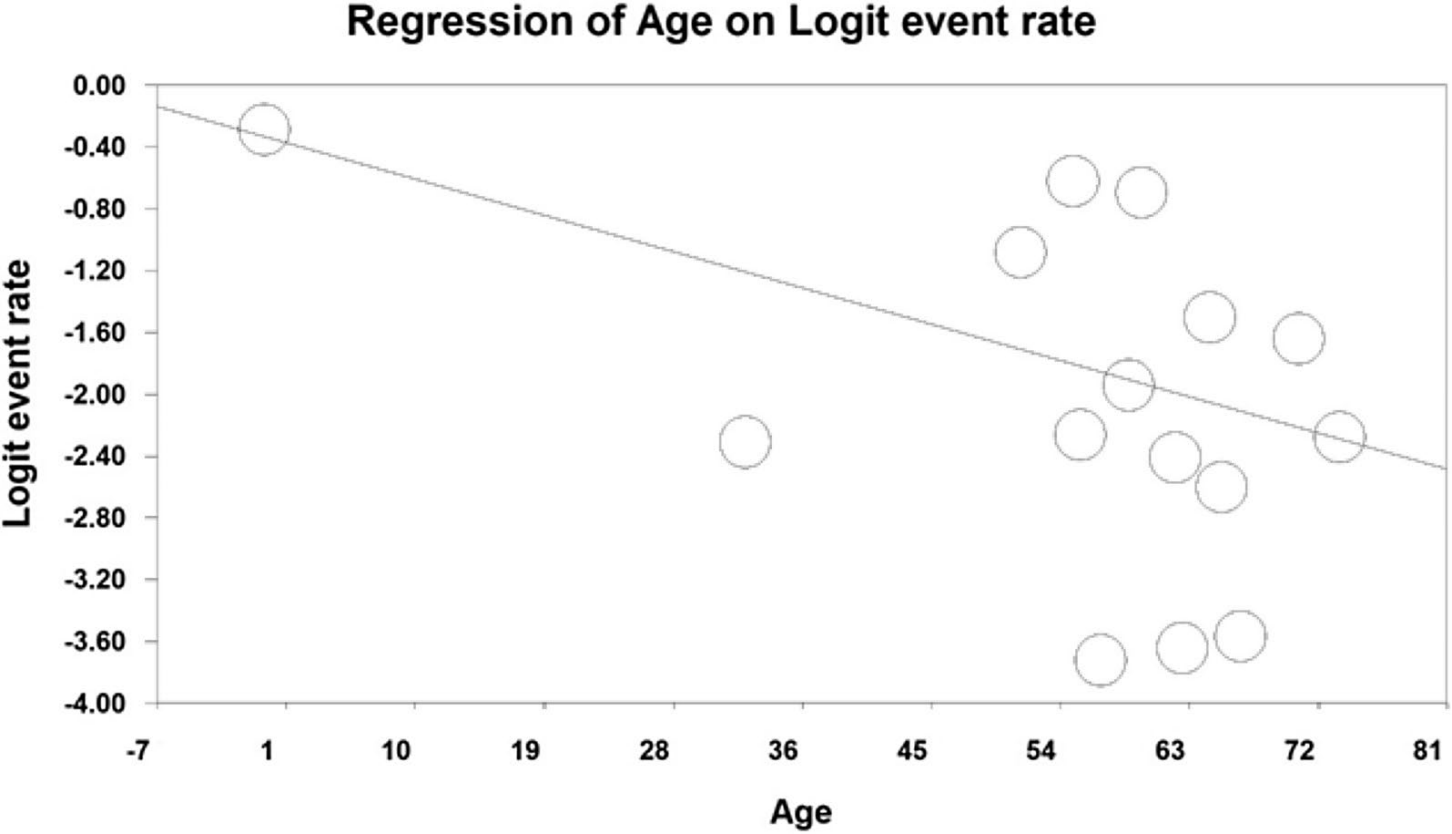
Meta-regression between participants' age in the study and prevalence of urinary tract infections in type 2 diabetic patients

Using meta-regression, the relationship between year of study and sample size and age of participants in the study with the prevalence of urinary tract infection in type 2 diabetic patients was examined. The prevalence of urinary tract infection was significantly different in all three cases. The prevalence of urinary tract infections in diabetic patients increased with increasing number of years of research, (*p* < 0.05), and with increasing age of participants (*p* < 0.05), but however the prevalence decreased with increasing sample size (*p* < 0.05) (Figs. 5, 6, 7).

## Discussion

According to the studies studied, the prevalence of urinary tract infections in patients with type 2 diabetes varies in different countries of the world. On average, 10.16% of patients with type 2 diabetes who participated in the study had a urinary tract infection, compared with 33.29% at Nicholas 2017 (conducted in the United States and Germany) and at Hirji 2012 (in the United States, Germany, and Sweden), with a prevalence rate of 2.55%, has the highest and lowest prevalence rates, respectively [29, 34]. People with type 2 diabetes have a higher chance of developing infections than non-diabetics [40, 41]. Providing accurate statistics can help health policy-makers make effective decisions and reduce costs and costs of health care. The prevalence reported in this study is an accurate value for policy and intervention measures.

A person with UTI is considered to have certain microbial pathogens in his or her urinary tract [42]. In people with type 2 diabetes, the main organism that causes urinary tract infections is the *E. coli* bacterium [3, 39, 43]. Bacterial urinary tract infections are clinically distinct. Septic bacteria cause urinary tract infections with apparent symptoms such as increased frequency of urination, dysuria, haematuria, and a painful touch of the hyperaemic area, while aseptic bacteria cause urinary tract infection without obvious symptoms [42]. It is said that the prevalence of aseptic bacteria in people with diabetes is three times higher than in normal people [44]. Also, asymptomatic bacterial infections are more common in these people, which does not indicate that their upper urinary tract is not involved. These people have been observed [45].

In people with type 2 diabetes, several different mechanisms may increase the risk of urinary tract infections, including diabetic nephropathy, autonomic neuropathy, immune system disorders, and glucosuria [9, 46].

Diabetic nephropathy leads to disorders such as protein excretion and severe glucosuria. Neurological damage associated with high blood sugar levels can adversely affect the ability of the bladder sensation. Sensory bladder sensory disturbances cause urinary retention, and increases urinary tract infections [47–51].

Diabetes reduces blood circulation, so as diabetes lengthens, it weakens the immune system, which is reduced by treating certain cytokines such as IL_6 and other anti-inflammatory cytokines in a diabetic patient. On the other hand, there are abnormal leukocytes. In diabetics and impaired phagocytic function, leukocytes due to high glucose levels in diabetic patients may weaken the immune system of these patients [52–56].

Apart from BMI, UTI is significantly associated with age, sex, recent UTI history and microalbuminuria [57].

It should be noted that there is a difference of opinion regarding the effect of diabetes duration and blood sugar control on UTI. The study of Vismanthan linked the duration of diabetes to UTI, but this was in contrast to findings by He 2018 [26, 36]. The relationship between blood sugar control and UTI is also highly controversial. It is effective in UTI, but in Greeling’s study, blood sugar control did not affect whether or not UTI was present [57–59].

With increasing age, the risk of developing UTI in both sexes, especially in women, increases. For example, in the Carrondo study, the UTI rate in people aged 18–64 was 9%, compared with 27.5% in people over 85 years old [27]. In all of the articles reviewed, the UTI rate in women was higher than in men, which appears to be related to bladder neurological dysfunction, physiological bladder changes due to aging or shortness of breath, and proximity to the anus among women [24, 27].

For example, a study by Carrondo 2020 in Portugal found that 23.6% of women with type 2 diabetes had UTI, compared to only 10.5% of men with type 2 diabetes [27]. A 2011 study in Fu2014 [11] reported a 14% increase in UTI incidence in women with type 2 diabetes and 9.1% in non-diabetic women, compared with 5% in men with type 2 diabetes and 2.4% in non-diabetic men.

The association between diabetes, urinary tract infection and gender has been well established [11]. In a 2018 study in China, out of 1072 women with type 2 diabetes in the study, 341 people were infected, and of the 1783 men with type 2 diabetes in the study, only 68 people had a urinary tract infection [28]. In connection with the study of Venmans, where the prevalence rate of UTI in men is higher than in women, it is necessary to provide the necessary explanations. In this study, the prevalence rate of recurrent bacterial cystitis in women was 2%, while in men, the prevalence of bacterial cystitis and prostatitis was 3%, so this could be a possible cause of the discrepancy [37].

Therefore, being female can be considered a risk factor for urinary tract infection. The prevalence of UTI in Stage 1 diabetics is higher than in Stage 2, because Stage 2 diabetics already have blood sugar control [8, 60].

For example, in the Carrondo 2020 study, the prevalence rate of UTI in diabetic patients was Stage 1, 24.4%, and in diabetic patients, Stage 2, was 4.8% [27]. In an epidemiological study, the prevalence of urinary tract infections was highest in developing countries (24%) and 12.9% and 19.6% in the United States and Europe, respectively [61]. One of the limitations of this study, which is mainly due to the review of the study, is the following:

1. Not all articles are available.
2. The method of measuring the variables studied is not the same in all studies.
3. Ignoring nutrition and lifestyle in all studies.
4. Due to the inconsistency of the study conditions and the volume of the samples, it is not possible to generalise the results of the present study.
5. It is hoped that the present study provides an organised and complete perspective for developing screening programs, appropriate planning, and health care policies to prevent the increase in the incidence and complications of UTI in people with type 2 diabetes.

## Conclusion

This study shows that urinary tract infections are highly prevalent in patients with type 2 diabetes. Therefore, due to the growing prevalence of diabetes and its complications, such as urinary tract infections, the need for appropriate screening programs and health care policies is becoming more apparent.

## Data Availability

Datasets are available through the corresponding author upon reasonable request.

## Abbreviations

UTI: Urinary tract infection
SID: Scientific Information Database
WoS: Web of Science
PRISMA: Preferred Reporting Items for Systematic Reviews and Meta-Analysis
STROBE: Strengthening the Reporting of Observational Studies in Epidemiology for cross-sectional Study
